# Structural basis for activation of CB1 by an endocannabinoid analog

**DOI:** 10.1038/s41467-023-37864-4

**Published:** 2023-05-09

**Authors:** Kaavya Krishna Kumar, Michael J. Robertson, Elina Thadhani, Haoqing Wang, Carl-Mikael Suomivuori, Alexander S. Powers, Lipin Ji, Spyros P. Nikas, Ron O. Dror, Asuka Inoue, Alexandros Makriyannis, Georgios Skiniotis, Brian Kobilka

**Affiliations:** 1 Department of Molecular and Cellular Physiology, Stanford University School of Medicine, 279 Campus Drive, Stanford, CA 94305 USA; 2 Department of Structural Biology, Stanford University School of Medicine, 279 Campus Drive, Stanford, CA 94305 USA; 3 Department of Computer Science, Stanford University, Stanford, CA 94305 USA; 4 Institute for Computational and Mathematical Engineering, Stanford University, Stanford, CA 94305 USA; 5 Department of Chemistry, Stanford University, Stanford, CA 94305 USA; 6 Center for Drug Discovery and Department of Pharmaceutical Sciences, Northeastern University, Boston, MA 02115 USA; 7 Graduate School of Pharmaceutical Sciences, Tohoku University, Sendai, Miyagi 980-8578 Japan; 8 Department of Chemistry and Chemical Biology, Northeastern University, Boston, MA 02115 USA; 9 Department of Photon Science, SLAC National Accelerator Laboratory, Stanford University, Menlo Park, CA 94025 USA

**Keywords:** Cryoelectron microscopy, G protein-coupled receptors

## Abstract

Endocannabinoids (eCBs) are endogenous ligands of the cannabinoid receptor 1 (CB1), a G protein-coupled receptor that regulates a number of therapeutically relevant physiological responses. Hence, understanding the structural and functional consequences of eCB-CB1 interactions has important implications for designing effective drugs targeting this receptor. To characterize the molecular details of eCB interaction with CB1, we utilized AMG315, an analog of the eCB anandamide to determine the structure of the AMG315-bound CB1 signaling complex. Compared to previous structures, the ligand binding pocket shows some differences. Using docking, molecular dynamics simulations, and signaling assays we investigated the functional consequences of ligand interactions with the “toggle switch” residues F200^3.36^ and W356^6.48^. Further, we show that ligand-TM2 interactions drive changes to residues on the intracellular side of TM2 and are a determinant of efficacy in activating G protein. These intracellular TM2 rearrangements are unique to CB1 and are exploited by a CB1-specific allosteric modulator.

## Abstract (web summary)

This manuscript describes the structure of an endocannabinoid analog-bound CB1 complex and reveals the structural determinants of ligand efficacy. The activation mechanism, unique to CB1, that is exploited by allosteric modulators is also outlined.

## Introduction

The cannabinoid receptor type 1 (CB1) is a critical component of the endocannabinoid system and the most abundantly expressed G protein-coupled receptor (GPCR) in the brain^1^. As CB1 regulates a wide range of neuronal functions, it is an attractive target for treating pain, anxiety, anorexia, and neurodegenerative disorders^2–4^. CB1 is activated by two endogenous cannabinoids (eCBs), arachidonoyl ethanolamine (anandamide) and 2-arachidonoyl-*sn*-glycerol (2-AG), that are derivatives of arachidonic acid^5^. CB1 is also activated by many structurally diverse exogenous ligands, most notably, the plant-derived classical cannabinoid (-)-Δ^9^-tetrahydrocannabinol (Δ^9^-THC), the non-classical cannabinoids, exemplified by CP-55940, and synthetic cannabinoid receptor agonists (SCRAs) that have emerged as illicit, designer drugs of abuse^6^. Apart from orthosteric agonists, allosteric modulators of CB1 have also been developed^7^.

Despite their promising therapeutic potential, exogenous CB1 agonists elicit adverse effects including severe agitation, anxiety, nausea, vomiting, tachycardia, elevated blood pressure, tremors, seizures, and hallucinations^6^. Moreover, chronic CB1 activation by orthosteric agonists leads to tolerance and dependence^8^. In addition to these adverse effects, SCRA use is associated with more severe adverse effects that may even result in death^8^. There is increasing evidence that these severe adverse effects caused by SCRAs could be a result of the super-efficacious activation of CB1 signaling that might lead to erratic neurotransmitter modulation and toxicity. In contrast, positive allosteric modulators (PAMs) of CB1 have shown efficacy in enhancing the antinociceptive effects of endocannabinoids in vivo without the adverse effects or tolerance noted above^9^.

At the cellular level, CB1 predominantly signals through the adenylate cyclase inhibitory G protein family, G_i/o_, and also recruits arrestins^10^. Different ligands may differentially stabilize conformations that favor interaction with specific G_i/o_ subtypes and downstream effectors^11^. Ligands that bias the receptor towards interactions with specific G_i/o_ subtypes or arrestins may exhibit different behavioral outcomes, thus modulating the therapeutic window^12^. Therefore, a better understanding of the structural basis of CB1 activation with diverse ligands could offer valuable insight and enhance our ability to design novel drugs with improved pharmacological profiles.

We have previously determined the structure of CB1 bound to the SCRA, MDMB-Fubinaca (FUB)^13^ and others have determined CB1 structures bound to the classical cannabinoid analogs AM841^14^ and AM11542^15^, the non-classical cannabinoid CP-55,940 and the negative allosteric modulator (NAM) Org27569^16^. However, no structure of CB1 bound to an eCB is available. To define the structural basis of CB1 activation by eCBs, we determined a cryo-EM structure of CB1 bound to the eCB analog AMG315 in a complex with heterotrimeric G_i_1 protein. To gain a better understanding of the downstream pathways activated by the ligands (eCBs, phytocannabinoids, and synthetic cannabinoids), we used fluorescence spectroscopy and signaling assays to show that different cannabinoids activate G_i_1 to different extents. Further, with molecular dynamics (MD) simulations, mutagenesis and signaling data we provide insights into the structural determinants of ligand efficacy in CB1.

## Results

### Activation of G_i_1

**Fig. 1:**
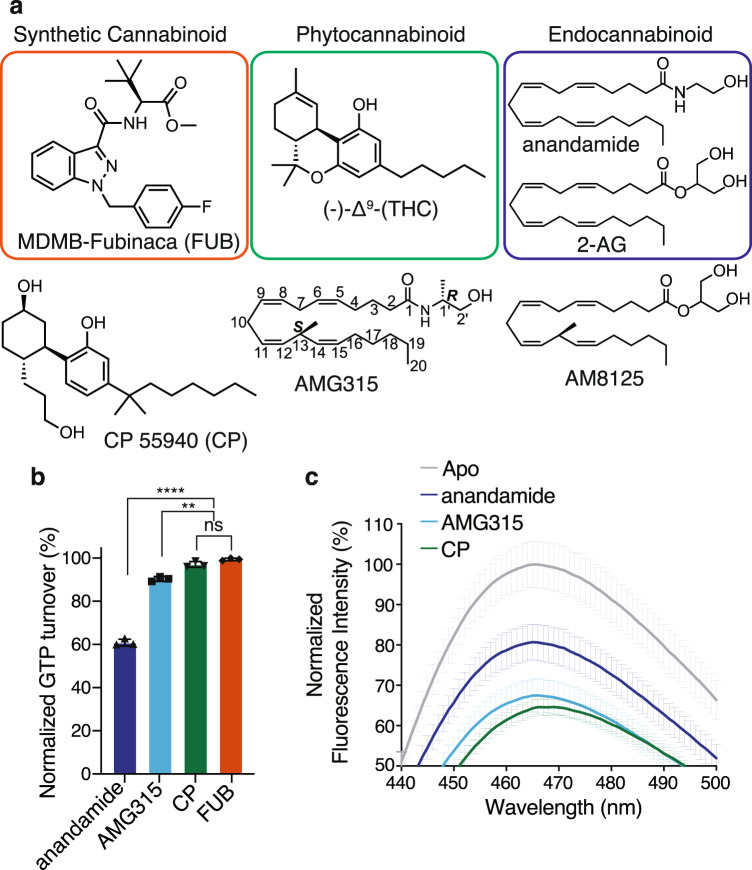
CB1 activation and GTP turnover by cannabinoids. **a** Chemical structures of a synthetic cannabinoid (MDMB-Fubinaca, FUB), a phytocannabinoid ((−)-*trans*-Δ^9^-tetrahydrocannabinol, (-)Δ^9^-THC) and the endocannabinoids (anandamide and 2-Arachidonoylglycerol, 2-AG). Structures of analogs of phytocannabinoid (CP 55940, CP) and endocannabinoid (AMG315 and AM8125) were used in this study. **b** GTP turnover assay showing efficient turnover produced by CP (CP 55940), AMG315 and anandamide with G_i_1 (Data normalization done with FUB (MDMB-Fubinaca) as 100% and receptor alone as 0%). Data represented as mean ± SD, *p* < 0.0001****, *p* = 0.0026** and *p* = 0.0507 (ns), unpaired *t*-test (two-tailed), *n* = 3 independent). **c** Bimane spectra monitoring TM6 showing differences between anandamide-bound CB1 compared to CP (CP 55940) and AMG315 (Data represented as mean ± SD, *n* = 3 independent experiment).

CB1 preferentially signals via G_i/o_ G protein subtypes. To investigate how structurally diverse ligands activate G_i_1, we performed a GTP turnover assay using the non-classical cannabinoid CP-55,940 (CP), the synthetic cannabinoid FUB, the eCB anandamide, as well as AMG315, an eCB analog that has two carefully chosen chiral centers and exhibits remarkable biological activity and stability^17^ (Fig. 1a). The full agonists CP and FUB were equally efficacious towards G_i_1 (Fig. 1b). When compared to CP and FUB, AMG315 was slightly less efficacious for G_i_1 (Fig. 1b). In contrast, anandamide acted as a partial agonist for G_i_1 (Fig. 1b) inducing only 60% of the GTP turnover in G_i_1 compared to CP and FUB.

To understand how ligands of different efficacies stabilize TM6, we performed fluorescence spectroscopy with CB1 labeled with the environmental-sensitive fluorophore, monobromobimane (bimane) as a conformational reporter of TM6 activation of CB1. To enable site-specific labeling, a minimal cysteine version of CB1 was generated^18^ where all the cysteine residues (except C256 and C264 which form a disulfide) were mutated to alanine. A cysteine residue was engineered at residue 341 (L6.33) on TM6, which was labeled with bimane. The bimane spectra of CP- and AMG315-bound CB1 were not significantly different except for a small (1 nm) blue-shift in *λ*_max_ (Fig. 1c). Adding anandamide to CB1 results in a smaller decrease in intensity and a blue-shift in *λ*_max_ by 4 nm compared to CP and 3 nm compared to AMG315 (Fig. 1c). These differences in the bimane spectra suggest that anandamide may stabilize a distinct conformation in TM6.

### Determination of an endocannabinoid-bound CB1–G_i_1 complex

**Fig. 2:**
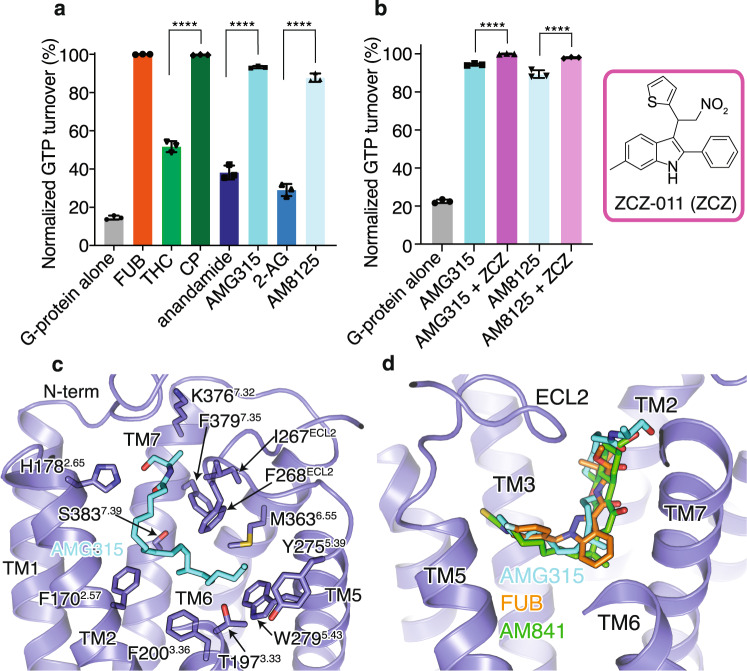
Endocannabinoid analog, AMG315-bound CB1–G_i_1 structure. **a** GTP turnover assay with G_i_1 showing maximum turnover produced by FUB (MDMB-Fubinaca) and CP (CP 55940). Endocannabinoid produce much lower GTP turnover compared to their analogs. Data represented as mean ± SD, *p* < 0.0001****, unpaired *t*-test (two-tailed), *n* = 3 independent experiments). **b** Addition of the PAM, ZCZ increases GTP turnover of the endocannabinoid analogs. (mean ± SD, *p* < 0.0001****, unpaired *t*-test (two-tailed), *n* = 3 independent experiments). **c** AMG315 binding pocket showing residues that are within 4 Å from the ligand. **d.** Overlay of ligands from different chemical classes, synthetic cannabinoid (FUB, orange), classical cannabinoid (AM841, green), and endocannabinoid (AMG315, blue).

To better understand the structural differences in the ligand-binding mode of eCBs compared to SCRAs like FUB^13^ and the classical cannabinoid AM841^14^, we sought to determine the structure of an eCB analog-bound CB1 signaling complex. To this end, we tested eCB analogs of anandamide and 2-AG for their ability to induce CB1-dependant G_i_1 GTP turnover as a measure of complex formation and stability. The anandamide analog AMG315 and the 2-AG analog AM8125 (Fig. 1a) induced significantly better GTP turnover compared to their parent compounds (Fig. 2a). However, as expected, neither AMG315 nor AM8125 was more efficacious than FUB or CP (Fig. 2a). Size-exclusion chromatography (SEC) showed that CB1 formed a slightly more stable complex with AMG315 than AM8125 (Supplementary Fig. 1a). The PAM ZCZ-011 (ZCZ) was able to further improve GTP turnover and further stabilize the complex (Fig. 2b). CB1 bound to AMG315 and ZCZ formed a complex with G_i_1 that was stable enough for cryoEM imaging yielding a density map at a global nominal resolution of 2.8 Å (Supplementary Fig. 1b). To improve the map densities in the region of interest, we performed local refinement focused on the receptor and ras-like domain of G_i_, yielding a map of indicated 3.2 Å resolution but with improved features for modeling. Accordingly, we created a composite map from the global and local maps to generate the full model used for our analysis.

Recently, structures of CB1 bound to ZCZ were determined, showing a binding site involving the intracellular ends of TMs 2, 3, and 4^19^. However, we were unable to see any density in the region described in this previous study^19^, and hence we do not model ZCZ in our structure. Overall, the mode of G_i_ engagement with the endo-bound CB1 is very similar to the previously determined structure of the FUB-bound CB1 complex (Supplementary Fig. 2a).

### AMG315 interactions with CB1

The cryoEM map shows well-defined density that allows unambiguous modeling of AMG315 and all the protein components of the CB1–G_i_1 complex (Supplementary Fig. 1b, Supplementary Fig. 2b). AMG315 engages the receptor through hydrophobic and polar interactions (Fig. 2c, Supplementary Fig. 2c). The acyl chain of AMG315 is buried deep in the binding pocket, while the polar head group is closer to the extracellular pocket, interacting with the ‘lid-like’ N-terminus (Fig. 2c) and pointing into a largely positive cavity formed by the TM1–TM7 interface (Supplementary Fig. 2d). Previously we observed a phospholipid molecule bound in the TM1–TM7 interface during MD simulations^13^. Phospholipids are the precursors for endocannabinoids and lipid binding observed in the simulations might indicate the ligand entry point for endocannabinoid through the membrane. The ligand access point in the TM1–TM7 interface is positively charged while the rest of the binding pocket is largely uncharged (Supplementary Fig. 2d). This charge distribution might help align the acyl chain and the hydroxyl of the endocannabinoid head group and guide the ligand correctly into the binding pocket. This mechanism of guided ligand entry has been proposed for other lipid-binding GPCRs such as S1P1 and LPA1^20^.

AMG315 overlays well with the previously determined structures of distinct classes of cannabinoids such as FUB^13^ and AM841^21^ (Fig. 2d). In addition to most of the hydrophobic and polar interactions made by FUB and AM841, AMG315, through its carbonyl head group, interacts with residues, F268^ECL2^ and I267^ECL2^ in ECL2 (Fig. 2c). When comparing CB1 and CB2, the N-terminus and ECL2 are the most diverse regions in terms of length and sequence conservation. The interactions of AMG315 with these regions might explain its 20-fold selectivity for CB1 over CB2^17^.

**Fig. 3:**
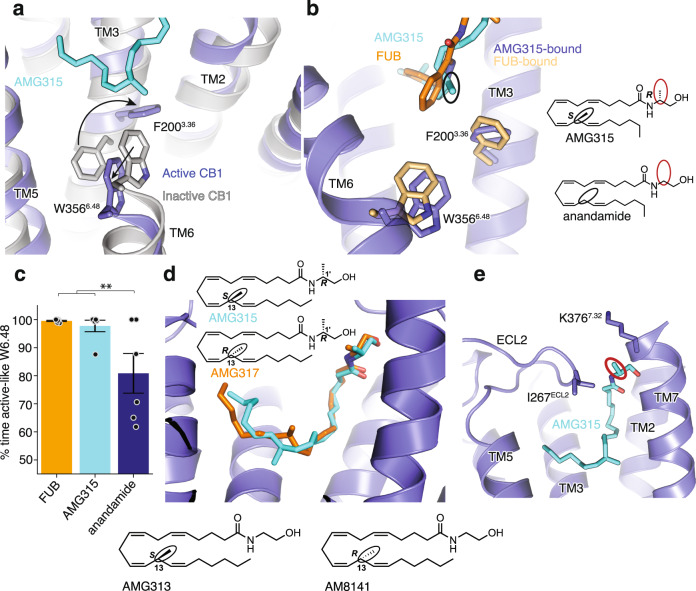
CB1 “Toggle switch” interaction with ligands. **a** AMG315 stabilizes the ‘toggle switch’ residues W356^6.48^ and F200^3.36^ in the active state. **b** Ligand interaction with residues W356^6.48^ and F200^3.36^. The methyl group in AMG315 (circled black) not present in anandamide interacts with the ‘toggle switch’. **c** In the simulation, anandamide stabilizes active-like conformations of W356^6.48^ significantly less when compared individually to FUB (MDMB-Fubinaca) (*p* = 0.02, two-sided Welch’s *t*-test) and to AMG315 (*p* = 0.04), as well as when compared to both FUB (MDMB-Fubinaca) and AMG315 as a group (*p* = 0.003). Data are presented as mean values ± SEM from *n* = 6 independent simulations (**p < 0.01). **d** Molecular docking shows that the R-stereochemistry (AMG317) instead of the S-stereochemistry at position 13 (AMG315), repositions the methyl group away from the “toggle switch”. Insert below: chemical structures of 13-methyl substituted anandamide analogs, AMG313 (13S-enantiomer) and AM8141 (13R-enantiomer). **e** The methyl group at position 1’ (circled red in 3B) on AMG315 interacts with residues on ECL2 (Ile267^ECL2^) and the extracellular region of TM7 (K376^7.32^). This substitution is not found in anandamide.

The residues, F200^3.36^ and W356^6.48^ (known as the “toggle switch”) play an important role in stabilizing the inactive conformation of CB1^22^, wherein F200^3.36^ and W356^6.48^ form π–π aromatic stacking interactions (gray, Fig. 3a). Additionally, the F200^3.36^A mutation has been shown to increase the basal activity of CB1^22^. Since these two residues are important for CB1 signaling activity, we postulated that a ligand’s efficacy correlates with its ability to engage the “toggle switch” to activate CB1. Upon activation, the rotation of TM3 and TM6 disrupts the stacking of F200^3.36^ and W356^6.48^ (Fig. 3a), with the phenyl ring of F200^3.36^ pointing towards the ligand to form hydrophobic interactions. In the FUB-bound structure, F200^3.36^ interacts with the ligands’ indazole ring (Fig. 3b). In the case of AMG315, the methyl group at C-13 (S-stereochemistry) interacts with the “toggle switch” residues (Fig. 3b, black circle). Molecular dynamic (MD) simulations that we performed show that anandamide stabilizes W356^6.48^ in the active-like conformation significantly less than FUB and AMG315 (Fig. 3c, Supplementary Fig. 2e–h), implying that the lower efficacy of anandamide might be related to its weaker interactions with the “toggle switch” residues. Molecular docking shows that using the diastereomer AMG317 with the R-stereochemistry at C-13 instead of S-stereochemistry as seen in AMG315, makes the methyl substitution point away from the “toggle switch” (Fig. 3d), and consequently no detectable receptor activation is observed for AMG317 (R-stereochemistry, Fig. 3d)^17^. Furthermore, by comparing anandamide with its (S)-C-13 methyl congener (AMG313, Fig. 3d), we observe that this methyl group imparts a 5-fold increase in potency and an increase in efficacy. However, the (R)-C-13 methyl enantiomer, AM8141 (Fig. 3d), shows no detectable activity at CB1^17^. The other methyl substitution (Fig. 3e, red circle) on AMG315 compared to anandamide at C-1’ interact with residues on ECL2 (Ile267^ECL2^) and the extracellular region of TM7 (K376^7.32^) (Fig. 3e). The combined interactions of the receptor with AMG315 due to the two chiral methyl groups synergize to result in an increase in potency of over 100-fold compared to anandamide^17^.

### Role of TM2 in ligand efficacy

**Fig. 4:**
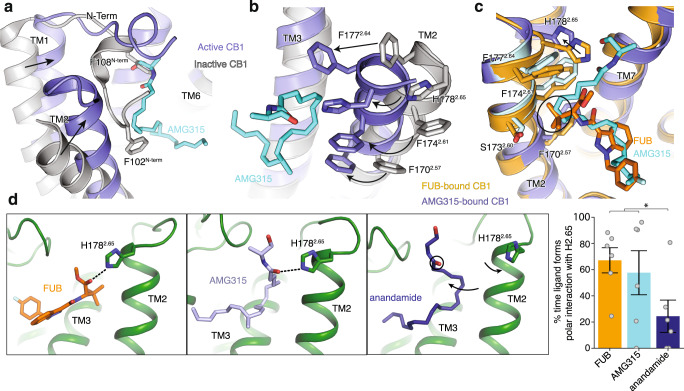
Ligand–TM2 interaction. **a** Overlay of inactive structure of CB1 (PDB: 5U09, white) and AMG315-bound structure (blue) showing the inward movement of TM2 upon activation and displacement of the N-terminus. **b** The inward movement of TM2 from the inactive state (gray) to the active state (blue) results in the translocation of residues F177^2.64^, H178^2.65^, F174^2.61^, and F170^2.57^ towards the agonist. **c** FUB (MDMB-Fubinaca) interacts with more TM2 residues (orange) compared to AMG315 (blue). F177^2.64^ and F174^2.61^ interact with FUB but not AMG315, shown in light blue. **d** In simulation, FUB (MDMB-Fubinaca) and AMG315 form polar interactions with H178^2.65^ more often than the less efficacious partial agonist anandamide (*p* = 0.03, two-sided Welch’s *t*-test, *p < 0.05). Data are presented as mean values ± SEM from *n* = 6 independent simulations.

Agonist interaction with TM2 appears to play an important role in CB1 activation. As with previous agonist-bound structures, the AMG315-bound CB1 shows extensive structural rearrangements in the ligand binding pocket compared to the antagonist-bound structure^21, 23^. Upon AMG315 binding, the N-terminus of CB1 is displaced from the transmembrane core, followed by the inward displacement of TM1 and TM2 (Fig. 4a). This inward movement of TM2 is accompanied by the repositioning of residues F170^2.57^, F174^2.61^, F177^2.64^, and H178^2.65^, that rotate towards and interact with the agonist (Fig. 4b). This repositioning has also been seen in the previous structures of agonist-bound CB1^13^. These structural differences in the ligand binding pocket between the binding of agonist and antagonist are not observed in the closely related CB2 receptor^14^^,^^24^ (Supplementary Fig. 3a). Since TM2 rearrangement is stabilized by agonist binding and mutations of these TM2 aromatic resides have been shown to affect ligand–receptor interactions^21^, these differences are an important part of ligand efficacy in CB1. In the previously determined FUB-bound CB1–G_i_1 structure, the *tert*-butyl group of FUB interacts with these repositioned residues on TM2 (Fig. 4c). MMB-Fubinaca, which has an isopropyl substitution at this position, has a reduced efficacy (Supplementary Fig. 3b) and potency^25^ compared to the *tert*-butyl substituent of FUB indicating that, in addition to interacting with the “toggle switch” residues, TM2-ligand interactions are an important determinant of ligand efficacy. Though AMG315 overlays well with FUB in the ligand binding pocket and makes similar interactions with the receptor, AMG315 is a less efficacious ligand compared to FUB. This difference in efficacy might be attributed in part to the interactions the ligands make with TM2, wherein FUB has more extensive interactions with TM2 than does AMG315 (residues that are further than 4 Å for the AMG315-bound structure are shown as light blue, Fig. 4c). The residues on TM2 that are within 4 Å of FUB are F170^2.57^, S173^2.60^, F174^2.61^, F177^2.64^ and H178^2.65^ (Fig. 4c). However, only residues F170^2.57^ and H178^2.65^ are within 4 Å of AMG315 (Fig. 4c). Studies have shown that adding a methyl substitution in anandamide at C-7 (AM11604, Supplementary Fig. 3c) increases the efficacy (*E*_max_) to 100% relative to the full agonist CP55940^17^, presumably due to its enhanced interactions with residues of TM2.

Compared to the FUB-bound structure, in the AMG315-bound CB1, H178^2.65^ has moved away from the ligand by ~2.5 Å (Fig. 4c). To investigate if there is a correlation between ligand efficacy and interaction with H178^2.65^, we performed MD simulations to probe the frequency of interactions between ligands (FUB, AMG315, and anandamide) and H178^2.65^. The full agonist FUB and the slightly less efficacious AMG315 more often form a polar interaction with H178^2.65^ compared to the partial agonist anandamide (Fig 4d). As described above, the methyl substitution at C-1’ in AMG315 interacts with K376^7.32^ and Ile267^ECL2^ which might limit its movement in the ligand binding pocket, increasing interaction frequency with H178^2.65^ (Fig. 4d). The absence of this methyl substitution in anandamide would allow the ligand to move away from TM2 and more often break interaction with H178^2.65^. Though not statistically significant, the frequency of polar interaction between H178^2.65^ and AMG315 is lower than with FUB (Fig. 4d).

### Distinct role of TM2 in activation of CB1

**Fig. 5:**
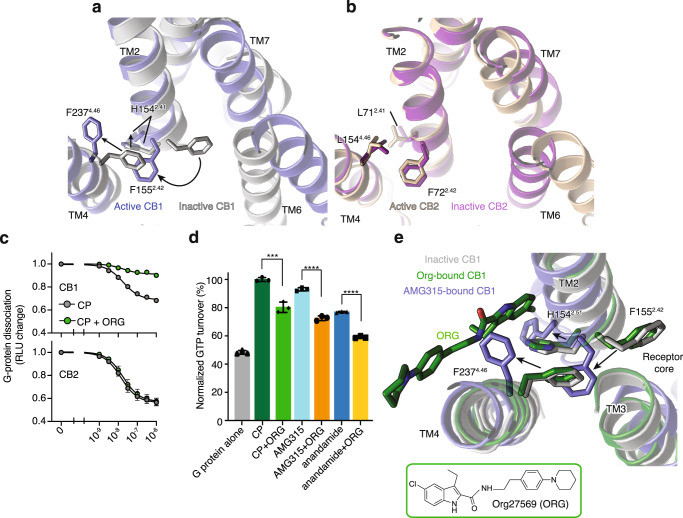
Structural changes in the intracellular side of TM2 upon CB1 activation. **a** Concerted movement of F237^4.46^ and F155^2.24^ upon activation of CB1 from inactive (inward, gray) to active (outward, blue) state. **b** The residue at position 4.46 in CB2 is a Leu and does not undergo movement upon activation. **c** NanoBiT-G-protein dissociation assay shows unchanged CP (CP 55940) response upon ORG (Org 27569) treatment in CB2. (mean ± SEM, *n* = 4 independent experiments). **d** GTP turnover assay showing reduced turnover in the presence of ORG (Org 27569) with G_i_1. (mean ± SD, *p* < 0.0001**** and *p* = 0.0010***, unpaired *t*-test (two-tailed), *n* = 3 independent experiments). **e** Structural rearrangement in F237^4.46^ and F155^2.42^ in ORG (Org 27569) (PDB: 6KQI, green) bound structure compared to active AMG315-bound (blue) and inactive (gray) structures. Residues F237^4.46^ and F155^2.42^ are inward towards the receptor core in the ORG-bound and inactive structures compared to AMG315-bound structure where they are outwards.

Changes in the extracellular end of TM2 upon agonist binding are associated with changes in the intracellular end of TM2, wherein a group of residues undergoes rearrangement upon activation. At the intracellular end of TM2, F155^2.42^ undergoes a concerted movement with F237^4.46^ upon activation. In the inactive structure, the aromatic ring of F237^4.46^ is facing inward, towards TM2-3, with F155^2.42^ positioned at the core of the receptor^23^ (Fig. 5a). Upon activation, F237^4.46^ and F155^2.42^ rotate outward away from the receptor core (Fig. 5a). Along with the F155^2.42^, the intracellular side of TM2 rotates with the sidechain of H154^2.41^ moving outward ~4 Å compared to the inactive CB1 structure (Fig. 5a).

In the inactive state, the receptor core facing F155^2.42^ interacts with TM3, the N^7.49^P^7.50^xxY^7.53^ motif and TM6^19^. These regions of the receptor (TM3, NPxxY, and TM6) undergo major changes upon activation, implying that the F155^2.42^ conformational change might be important for activation. Mutations to F155^2.42^ change ligand efficacy, with mutation to Trp decreasing efficacy and mutation to Ile increasing efficacy^19^. Metadynamics simulations data show that the highest energy barrier for CB1 transition from inactive (TM6 inward) to active (TM6 outward) state involves an intermediate, wherein F155^2.42^ changes its orientation before TM6 can move outward for full activation of the receptor^19^. This raises the possibility that a conformational change involving F155^2.42^ is a rate-limiting step for CB1 activation^19^. This contrasts with other receptors such as β_2_AR, μOR, and M2R (Supplementary Fig. 4a) where no structural change in residue at 2.42 is seen. Notably, this includes the closely related receptor, CB2 in which the corresponding residue F72^2.42^ is positioned outward both in the active^14^ and inactive^24^ structures.

F237^4.46^, the other residue involved in TM2 concerted movement, is unique in CB1 as other receptors do not have a bulky aromatic residue at position 4.46 (Supplementary Fig. 4a-b). Thus, unlike in other receptors, the outward movement of F155^2.42^ in CB1 will cause F237^4.46^ to orient outward to avoid a steric clash (Supplementary Fig. 4a, b). F237^4.46^ to a Leu (like in CB2) increases basal activity^26^.

Org27569 (ORG) is a CB1 selective negative allosteric modulator (NAM) (Fig. 5c) for CB1 with unusual pharmacology in that, unlike conventional NAMs, it increases agonist affinity^27^ while decreasing G_i_1 turnover (Fig. 5d). The effect of ORG on G protein activation can be explained by its binding to the interface of TM2 and TM4 and stabilizing the F155^2.42^-F237^4.46^ “activation switch” of CB1 in the inactive state (Fig. 5e). The mechanism by which ORG enhances agonist binding affinity has yet to be determined.

## Discussion

eCB signaling plays a critical role in maintaining homeostasis and is involved in the regulation of neurotransmission and synaptic plasticity^28^. Phytocannabinoids and synthetic cannabinoids that emulate eCB signaling through the CB1 receptor produce undesirable adverse effects^8^. Structurally and pharmacologically, eCBs are very distinct from phytocannabinoids and synthetic cannabinoids, and understanding signaling by eCBs has important implications for designing drugs with desired signaling profiles. Anandamide has a lower efficacy compared to the agonist CP and differences in the spectra of an environmentally sensitive fluorescent probe suggest that anandamide might stabilize a distinct conformation of the cytoplasmic end of TM6. To better understand the pharmacology of eCBs, we determined the cryoEM structure of a CB1-G_i_ signaling complex bound to AMG315, a metabolically stable and highly potent endocannabinoid analog. This compound interacts with the N-terminal, TM1 and TM7 regions of CB1 which are not exploited by other ligands. Using MD simulations and SAR data, we show that the efficacy of CB1 ligands depends on their propensity to interact with the ‘toggle switch’ residues F200^3.36^/W356^6.48^. Additionally, ligand efficacy in CB1 appears to be related to its interaction with the extracellular end of TM2. Ligand interactions in the extracellular region are transmitted to the intracellular end of TM2 where residue F155^2.42^ undergoes concerted movement with F237^4.46^ to contribute to the activation of CB1. This activation mechanism appears unique to CB1 (not seen in other GPCRs thus far) due to the distinctively positioned Phe residue at position 4.46.

## Methods

### Purification of CB1

CB1 was expressed and purified as described previously^13^. Briefly, human full-length CB1 containing an N-terminal FLAG tag and C-terminal histidine tag was expressed in *Spodoptera frugiperda Sf9* insect cells with the baculovirus method (Expression Systems). Receptor was extracted using 1% lauryl maltose neopentyl glycol (L-MNG) and purified by nickel-chelating Sepharose chromatography. The eluant from the Ni column was applied to an M1 anti-FLAG immunoaffinity resin. After washing to progressively decreasing the concentration of L-MNG, the receptor was eluted in a buffer consisting of 20 mM HEPES pH 7.5, 150 mM NaCl, 0.05% L-MNG, 0.005% cholesterol hemisuccinate (CHS), FLAG peptide and 5 mM EDTA. Finally, CB1 was purified with size exclusion chromatography, on Superdex 200 10/300 gel filtration column (GE) in 20 mM HEPES pH 7.5, 150 mM NaCl, 0.02% L-MNG, 0.002% CHS. Ligand-free CB1 was concentrated to ~500 µM and stored at −80 °C.

### Expression and purification of G_i_ heterotrimer

Heterotrimeric G_i_ was expressed and purified as previously described^29^. Insect cells (*Trichuplusia ni, Hi5*, Expression Systems) was co-infected with wild-type human Ga_i_ subunit virus and wild-type human b_1_g_2_ virus. b_1_g_2_ contains a histidine tag inserted at the amino terminus of the b subunit that is used for further purification. After harvesting cells expressing the heterotrimetric G-protein, they were lysed in a hypotonic buffer. Heterotrimeric G_i_b_1_g_2_ was extracted in a buffer containing 1% sodium cholate and 0.05% n-dodecyl-β-d-maltoside (DDM, Anatrace). Ni-NTA chromatography is performed and the detergent was exchanged from cholate/DDM to DDM on column. After elution, the protein was dialyzed overnight in 20 mM HEPES, pH 7.5, 100 mM sodium chloride, 0.1% DDM, 1 mM magnesium chloride, 100 μM TCEP and 10 μM GDP together with Human rhinovirus 3C protease (3C protease) to cleave off the amino-terminal 6xHis tag. 3C protease was removed by Ni-chelated sepharose and the heterotrimetric G-protein was further purified with MonoQ 10/100 GL column (GE Healthcare). Protein was bound to the column and washed in buffer A (20 mM HEPES, pH 7.5, 50 mM sodium chloride, 1 mM magnesium chloride, 0.05% DDM, 100 μM TCEP, and 10 μM GDP). The protein was eluted with a linear gradient of 0–50% buffer B (buffer A with 1 M NaCl). The collected G protein was dialyzed into 20 mM HEPES, pH 7.5, 100 mM sodium chloride, 1 mM magnesium chloride, 0.02% DDM, 100 μM TCEP, and 10 μM GDP. Protein was concentrated to 250 µM and flash frozen until further use.

### Purification of scFv16

scFv16 was purified with a hexahistidine-tag in the secreted form from *Trichuplusia ni Hi5* insect cells using the baculoviral method. The supernatant from baculoviral infected cells was pH balanced and quenched with chelating agents and loaded onto Ni resin. After washing with 20 mM HEPES pH 7.5, 500 mM NaCl, and 20 mM imidazole, the protein was eluted with 250 mM imidazole. Following dialysis with 3C protease into a buffer consisting of 20 mM HEPES pH 7.5 and 100 mM NaCl, scFv16 was further purified by reloading over Ni a column. The collected flow-through was applied onto a Superdex 200 16/60 column and the peak fraction was collected, concentrated, and flash-frozen.

### CB1–G_i_ complex formation and purification

CB1 in L-MNG was incubated with AMG315 and ZCZ (for all assay racemate ZCZ was used) for ~ 1 h at room temperature. Simultaneously, G_i_1 heterotrimer in DDM was incubated with 1% L-MNG at 4 °C. The AMG315- and ZCZ-bound CB1 was incubated with a 1.25 molar excess of detergent exchanged G_i_ heterotrimer at room temperature for ~3 h. To stabilize a nucleotide-free complex, apyrase was added and incubated for 1.5 h at 4 °C. The complex was diluted 4-fold with 20 mM HEPES pH 7.5, 100 mM NaCl, 0.8% L-MNG/0.08% CHS, 0.27% GDN/0.027% CHS, 1 mM MgCl_2_, 10 µM AMG315, 20 µM ZCZ, and 2 mM CaCl_2_ and purified by M1 anti-FLAG affinity chromatography. After washing to remove excess G protein and reduce detergents, the complex was eluted in 20 mM HEPES pH 7.5, 100 mM NaCl, 0.01% L-MNG/0.001% CHS, 0.0033% GDN/0.00033% CHS, 10 µM AMG315, 10 µM ZCZ, 5 mM EDTA, and FLAG peptide. The complex was supplemented with 100 µM TCEP and incubated with 2 molar excess of scFv16 overnight at 4 °C. Size exclusion chromatography (Superdex 200 10/300 Increase) was used to further purify the CB1–G_i_–scFv16 complex. The complex in 20 mM HEPES pH 7.5, 100 mM NaCl, 10 µM AMG315, 10 µM ZCZ, 0.00075% L-MNG/0.000075% CHS and 0.00025% GDN/0.000025% CHS was concentrated to ~15 mg/mL for electron microscopy studies.

### Bimane fluorescence

Minimal cysteine CB1 with 336C at 10 μM was incubated with 10-molar excess of bimane at room temperature for one hour. Excess label was removed using size exclusion chromatography on a Superdex 200 10/300 Increase column in 20 mM HEPES pH 7.5, 100 mM NaCl and 0.01% MNG/0.001% CHS. Bimane-labeled CB1 at 0.1 μM was incubated with ligands (10 μM) for one hour at room temperature. Fluorescence data were collected at room temperature in a 150 μL cuvette with a *FluorEssence v3.8 software* on a Fluorolog instrument (*Horiba*) in photon-counting mode. Bimane fluorescence was measured by excitation at 370 nm with excitation and emission bandwidth passes of 4 nm. The emission spectra were recorded from 410 to 510 nm with 1 nm increment and 0.1 s integration time.

### GTP turnover assay

Analysis of GTP turnover was performed by using a modified protocol of the GTPase-Glo^TM^ assay (Promega) described previously^30^. In the presence (20 μM) or absence of ligand, CB1 (1 μM) and G_i_ (1 μM) were mixed together in 20 mM HEPES, pH 7.5, 50 mM NaCl, 0.01% L-MNG, 100 μM TCEP, 10 μM GDP and 5 μM GTP and incubated at room temperature. GTPase-Glo-reagent was added to the sample after incubation for 60 min (agonist assays) or 30 min (for PAM assays). Luminescence was measured after the addition of detection reagent and incubation for 10 min at room temperature using a *SpectraMax Paradigm* plate reader.

### Cryo-EM data acquisition

For grid preparation, 3 μL of purified CB1–G_i_ complex at 15 mg/ml was applied on glow-discharged holey carbon gold grids (Quantifoil R1.2/1.3, 200 mesh). The grids were blotted using a Vitrobot Mark IV (FEI) with 4 s blotting time and blot force 3 at 100% humidity and plunge-frozen in liquid ethane. A total of 8332 movies were recorded on a Titan Krios electron microscope (Thermo Fisher Scientific-FEI) operating at 300 kV at a calibrated magnification of ×29,000 and corresponding to a pixel size of 0.8521 Å. Micrographs were recorded using a K3 Summit direct electron camera (Gatan Inc.) with a dose rate of 1.405 electrons/Å^2^/s. The total exposure time was 3.895 s with an accumulated dose of ~80.09 electrons per Å^2^ and a total of 57 frames per micrograph. Automatic data acquisition was done using *SerialEM*.

### Image processing and 3D reconstructions

Micrographs were imported into RELION 3.1 and beam-induced motion correction was performed with *MotionCor2* followed by CTF parameter fitting with *CTFFIND4*. Extracted particles were imported into *cryosparc* 3.3.1, sorted with iterative rounds of 2D classification followed by iterative rounds of 3D classification to arrive at a final curated stack of 530,918 particles. These particles were then imported back to RELION 3.1, subjected to Bayesian polishing^31^ and then brought back to *cryosparc* 3.3.1 for final reconstruction with nonuniform refinement and local refinement focused on the receptor and G_i_ ras domain. A composite map was generated from these two maps in *phenix* version 1.19.2 (Supplementary Fig. 1b).

### Model building and refinement

The initial template of CB1 was the FUB-bound CB1-G_i_ structure (PDB: 6N4B). Agonist coordinates and geometry restraints were generated using *phenix.elbow*. Models were docked into the EM density map using *UCSF Chimera*. Initial ligand placement was made with the *GemSpot* pipeline^32^. *Coot* was used for iterative model building and the final model was subjected to global refinement and minimization in real space using *phenix.real_space_refine* in *Phenix*. Model geometry was evaluated using *Molprobity*. FSC curves were calculated between the resulting model and the half map used for refinement as well as between the resulting model and the other half map for cross-validation (Supplementary Fig. 1b). The final refinement parameters are provided in Supplementary Table 1.

### MD simulations

#### System setup for MD simulation

We performed simulations of CB1R bound to the endocannabinoid anandamide, to the synthetic cannabinoid FUB, and to AMG315, an analog of anandamide. We initiated the simulations from the AMG315-bound structure (modeled into the non-composit map) that was solved in the presence of ZCZ presented in this paper. For all simulations, we removed the single chain variable fragment (scFv) and the G protein from the structure. For the FUB-bound and anandamide-bound simulations, we replaced the AMG315 molecule with FUB or anandamide in silico using Maestro (Schrödinger). To model anandamide, we edited the AMG315 molecule directly using the editor in Maestro (i.e., using the “build” panel). For FUB, we aligned the previously published FUB-bound structure (PDB: 6N4B)^13^ to the AMG315-bound structure, after which we replaced AMG315 with FUB. For each of these three simulation conditions, we performed six independent simulations in which initial atom velocities were assigned randomly and independently.

Neutral acetyl and methylamide groups were added to cap the N- and C-termini, respectively, of protein chains. Extracellular loop 2 (ECL2) loop of the receptor was modeled using the Maestro (Schrödinger) “Crosslink Proteins” tool, utilizing a fragment from the previously published structure of CB1 bound to agonist AM1 1542 (PDB: 5XRA)^15^. Titratable residues were kept in their dominant protonation states at pH 7, except for D2.50 (D163) and D3.49 (D213), which were protonated (neutral) in all simulations, as studies indicate that these conserved residues are protonated in active-state GPCRs^33, 34^. Histidine residues were modeled as neutral, with hydrogen bound to either the delta or epsilon nitrogen depending on which tautomeric state optimized the local hydrogen-bonding network. Dowser was used to add water molecules to protein cavities, and the protein structures were aligned on transmembrane (TM) helices of the FUB-bound active CB1 crystal structure (PDB: 6N4B)^13^ in the orientation of proteins in membranes (OPM) database^35^. The aligned structures were inserted into a pre-equilibrated palmitoyl-oleoyl-phosphatidylcholine (POPC) membrane bilayer using Dabble^36^. Sodium and chloride ions were added to neutralize each system at a concentration of 150 mM. Systems comprised 56,000 atoms, including ~140 lipid molecules and ~11,000 water molecules. Approximate system dimensions were 80 Å × 90 Å × 85 Å.

### Simulation protocols

Simulations were run using the AMBER18 software^37^ under periodic boundary conditions with the Compute Unified Device Architecture (CUDA) version of Particle-Mesh Ewald Molecular Dynamics (PMEMD) on graphics processing units (GPUs)^38^. The systems were first heated over 12.5 ps from 0 to 100 K in the NVT ensemble using a Langevin thermostat with harmonic restraints of 10.0 kcal mol^−1^ Å^−2^ on the non-hydrogen atoms of the lipids, protein, and ligand. Initial velocities were sampled from a Boltzmann distribution. The systems were then heated to 310 K over 125 ps in the NPT ensemble. Equilibration was performed at 310 K and 1 bar in the NPT ensemble, with harmonic restraints on the protein and ligand non-hydrogen atoms tapered off by 1.0 kcal mol^−1^ Å^−2^ starting at 5.0 kcal mol^−1^ Å^−2^ in a stepwise manner every 2 ns for 10 ns, and finally by 0.1 kcal mol^−1^ Å^−2^ every 2 ns for an additional 18 ns. All restraints were completely removed during the production simulation. Production simulations were performed at 310 K and 1 bar in the NPT ensemble using the Langevin thermostat and Monte Carlo barostat. The simulations were performed using a timestep of 4.0 fs while employing hydrogen mass repartitioning. Bond lengths were constrained using SHAKE. Non-bonded interactions were cut off at 9.0 Å, and long-range electrostatic interactions were calculated using the particle-mesh Ewald (PME) method with an Ewald coefficient (*β*) of ~0.31 Å and B-spline interpolation of order 4. The PME grid size was chosen such that the width of a grid cell was approximately 1 Å. We employed the CHARMM36m force field for protein molecules, the CHARMM36 parameter set for lipid molecules and salt ions, and the associated CHARMM TIP3P model for water^39, 40^. Ligand parameters were obtained using the CGenFF webserver^41, 42^. We validated these parameters as follows. First, we manually checked that the assigned atom types are accurate. Indeed, for all ligands in this paper, they are accurate. Second, CGenFF assigns penalties to ligand force-field parameters as a measure of uncertainty in the parameters; for high-penalty parameters, we performed further validation. For AMG315 and anandamide, all parameters have very low penalties. For FUB, a small number of high-penalty dihedral parameters were found. To validate the parameters for FUB, we first checked that the parameter analogies are reasonable (i.e., that the model compounds from which the analogy parameters are obtained are chemically similar to FUB), and indeed they are. For high-penalty flexible dihedrals, we also checked that the angle adopted in simulations is close to the experimentally determined cryo-EM structure with FUB, and, indeed, this was the case. We also checked the stability of FUB in the simulation. FUB overall is very stable in simulation, as seen from the ligand RMSD plots (Supplementary Fig. 2e), further supporting that the parameters are reasonable and consistent with the experimental data. However, one caveat is that we cannot rule out that the high-penalty dihedral parameters may lead to artificially high torsional barriers for FUB.

For each ligand, we performed six independent 2-µs simulations at 310 K. All simulations were performed on the Sherlock computing cluster at Stanford University.

### Simulation analysis protocols

The AmberTools17 CPPTRAJ package was used to reimage trajectories at 1 ns per frame, while visual molecular dynamics (VMD)^43^ was used for visualization and analysis. For all reported analyses, we discarded the first 0.5 µs of each simulation to achieve better equilibration.

For Fig. 3, we determined the fraction of time W6.48 (W356) spent in an active-like conformation by setting a threshold value of 6.7 Å for the distance between the beta carbon of W6.48 and the alpha carbon of C7.42 on TM7. Frames with a distance greater than the threshold were classified as active-like. To determine whether differences between simulations performed with different ligands were statistically significant, we performed two-sided *t*-tests of unequal variance (Welch’s *t*-test) on the frequency of this distance being above the threshold value, with each simulation as an independent sample.

For Fig. 4, we used GetContacts (https://getcontacts.github.io/) to determine the frequency of polar interactions between each ligand and H2.65 (H178) in the simulation. Specific polar contacts considered were direct hydrogen bonds or hydrogen bonds mediated by one water molecule. To determine whether differences between simulation conditions performed with different ligands were statistically significant, we performed two-sided *t*-tests of unequal variance (Welch’s *t*-test) on the frequency of polar interactions using each simulation as an independent sample.

### NanoBiT-G-protein dissociation assay

CB1-induced G-protein dissociation was measured by the NanoBiT-G-protein dissociation assay, in which the interaction between the Gα subunit and the Gγ subunit was monitored by the NanoBiT-based enzyme complementation system (Promega). Specifically, the NanoBiT-G_i_1 protein consisting of the Gαi1 subunit fused with a large fragment (LgBiT) at the α-helical domain and the N-terminally small fragment (SmBiT)-fused Gγ2 subunit was expressed, along with an untagged Gβ1 subunit and a test GPCR construct. CB1 construct with the N-terminal hemagglutinin signal sequence and the FLAG epitope tag with a flexible linker (MKTIIALSYIFCLVFADYKDDDDKGGSGGGGSGGSSSGGG) was inserted into the pCAGGS expression vector. HEK293A cells (Thermo Fisher Scientific) were seeded in a 10-cm culture dish at a concentration of 2 × 10^5^ cells ml^−1^ (10 ml per dish in DMEM (Nissui) supplemented with 10% fetal bovine serum (Gibco), glutamine, penicillin, and streptomycin), one day before transfection. The transfection solution was prepared by combining 25 µl (per dish hereafter) of polyethylenimine (PEI) Max solution (1 mg ml^−1^; Polysciences), 1 ml of Opti-MEM (Thermo Fisher Scientific), and a plasmid mixture consisting of 1 µg test GPCR construct, 500 ng LgBiT-containing Gαi1 subunit, 2.5 µg Gβ1 subunit and 2.5 µg SmBiT-fused Gγ2 subunit with the C68S mutant. After incubation for one day, the transfected cells were harvested with 0.5 mM EDTA-containing Dulbecco’s PBS, centrifuged, and suspended in 9 ml of HBSS containing 0.01% bovine serum albumin (BSA; fatty acid–free grade; SERVA) and 5 mM HEPES (pH 7.4) (assay buffer). The cell suspension was dispensed in a white 96-well plate at a volume of 70 µl per well and loaded with 20 µl of 50 µM coelenterazine (Carbosynth) diluted in the assay buffer. After a 2 h incubation at room temperature, the plate was measured for baseline luminescence (SpectraMax L, Molecular Devices) and a test allosteric ligand (10 µl) was manually added. The plate was immediately read at room temperature for the following 10 min as the kinetics mode, at measurement intervals of 20 s. Thereafter, a test orthosteric ligand (20 µl) was added and the plate was read for another 10 min. The luminescence counts over 3–5 min after ligand addition were averaged and normalized to the initial count. The fold-change values were further normalized to that of vehicle-treated samples and used to plot the G-protein dissociation response. Using the Prism 8 software (GraphPad Prism), the G-protein dissociation signals were fitted to a four-parameter sigmoidal concentration-response curve, from which pEC_50_ values (negative logarithmic values of EC_50_ values) and *E*_max_ values were used to calculate mean and SEM.

### Reporting summary

Further information on research design is available in the Nature Portfolio Reporting Summary linked to this article.

## Supplementary information

Supplementary Information

Reporting Summary

## Data Availability

The data that support this study are available from the corresponding authors upon request. The cryo-EM density maps have been deposited in the Electron Microscopy Data Bank (EMDB) under accession codes EMD-40052, EMD-40057, and EMD-40058. Model coordinates have been deposited in the Protein Data Bank (PDB) under accession number 8GHV. Raw cryo-EM micrographs have been deposited in the electron microscopy public image archive (EMPIAR) under the accession number EMPIAR-11474. Previously published structures can be accessed via accession codes: 6KQI, 5U09, 6PT0, 6KPC, 3SN6, 2RH1, 6DDE, 4DKL, 6OIK, 3UON, 5U09, 6N4B, 5XRA. Source data are provided with this paper.

## Source data

Source data
