# Supplementary material for: Structural basis for activation of CB1 by an endocannabinoid analog
Source: Nat Commun. 2023 May 9;14:2672. doi: 10.1038/s41467-023-37864-4 (PMC10169858; doi:10.1038/s41467-023-37864-4)
Supplement: Supplementary file 1 — Supplementary Information [file 41467_2023_37864_MOESM1_ESM.pdf]

Structural basis for activation of CB1 by an endocannabinoid analog

Kaavya Krishna Kumar[1]†, Michael J. Robertson[1,2]†, Elina Thadhani[1–4], Haoqing Wang[1], Carl-Mikael Suomivuori[1–4,], Alexander S. Powers[1–5], Lipin Ji[6], Spyros P. Nikas[6], Ron O. Dror[1–4], Asuka Inoue[7], Alexandros Makriyannis[6,8*], Georgios Skiniotis[1,2,9*], Brian Kobilka[1*]

[1]Department of Molecular and Cellular Physiology, Stanford University School of Medicine, 279 Campus Drive, Stanford, California 94305, USA.

[2]Department of Structural Biology, Stanford University School of Medicine, 279 Campus Drive, Stanford, California 94305, USA.

[3]Department of Computer Science, Stanford University, Stanford, California 94305, USA.

[4]Institute for Computational and Mathematical Engineering, Stanford University, Stanford, CA 94305, USA

[5]Department of Chemistry, Stanford University, Stanford, California 94305, USA

[6]Center for Drug Discovery and Department of Pharmaceutical Sciences, Northeastern University, Boston, MA 02115, USA.

[7]Graduate School of Pharmaceutical Sciences, Tohoku University, Sendai, Miyagi 980-8578, Japan.

[8]Department of Chemistry and Chemical Biology, Northeastern University, Boston, MA 02115, USA.

[9]Department of Photon Science, SLAC National Accelerator Laboratory, Stanford University, Menlo Park, California 94025, USA.

† Equal Contribution

*Correspondence: a.makriyannis@northeastern.edu (A.M.), yiorgo@stanford.edu (G.S.) and kobilka@stanford.edu (B.K.K)

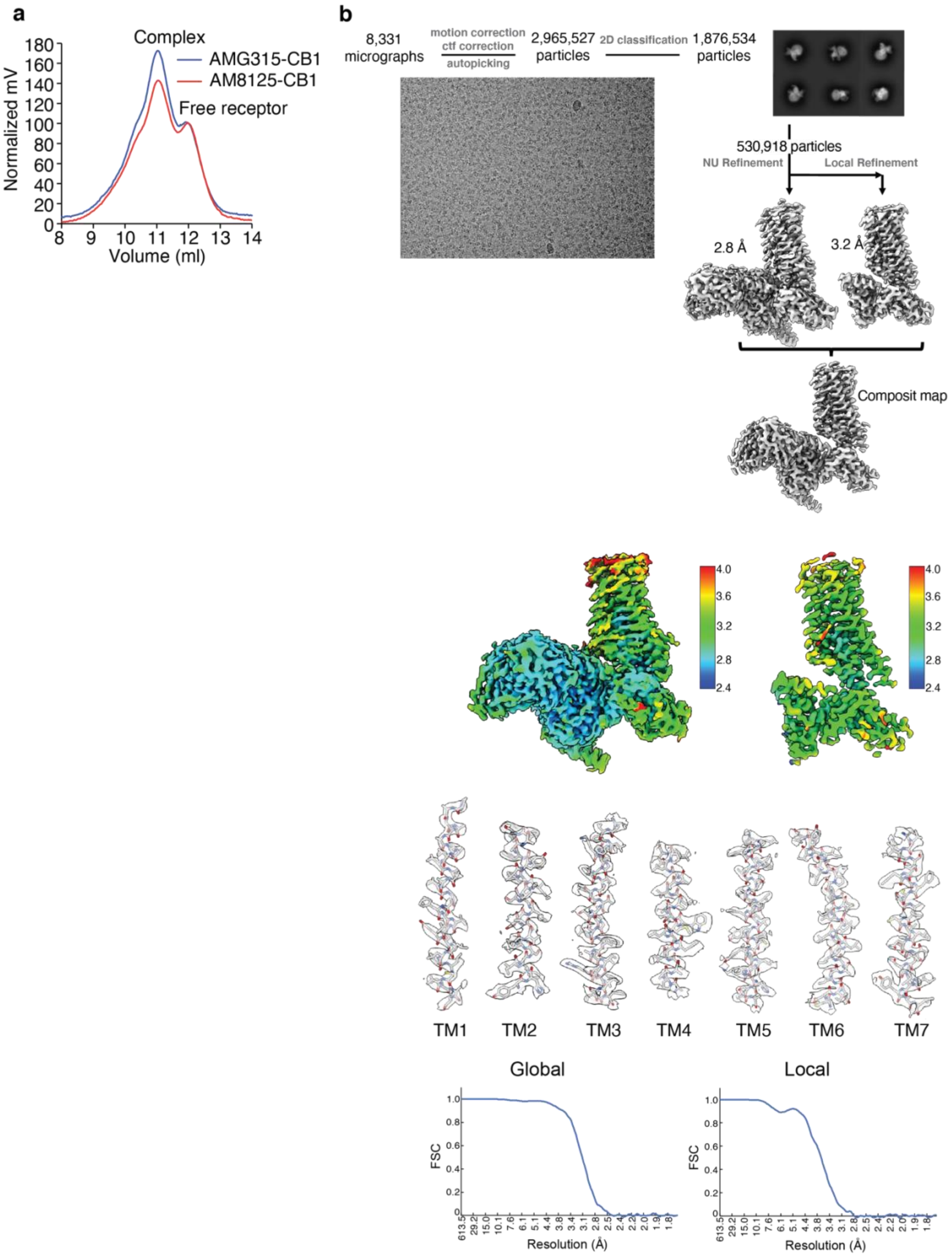

Supplementary Fig.1: Complex formation and cryo-EM data processing

a. Fluorescence-detection size exclusion chromatography (FSEC) traces showing complex peak and free receptor peak with the different AMG315 (blue) and AM8125 (red).
b. Cryo-EM processing flow chart, including particle selection, classification and density map reconstruction. Density of helices (TM1-7) and FSC curves are also shown.

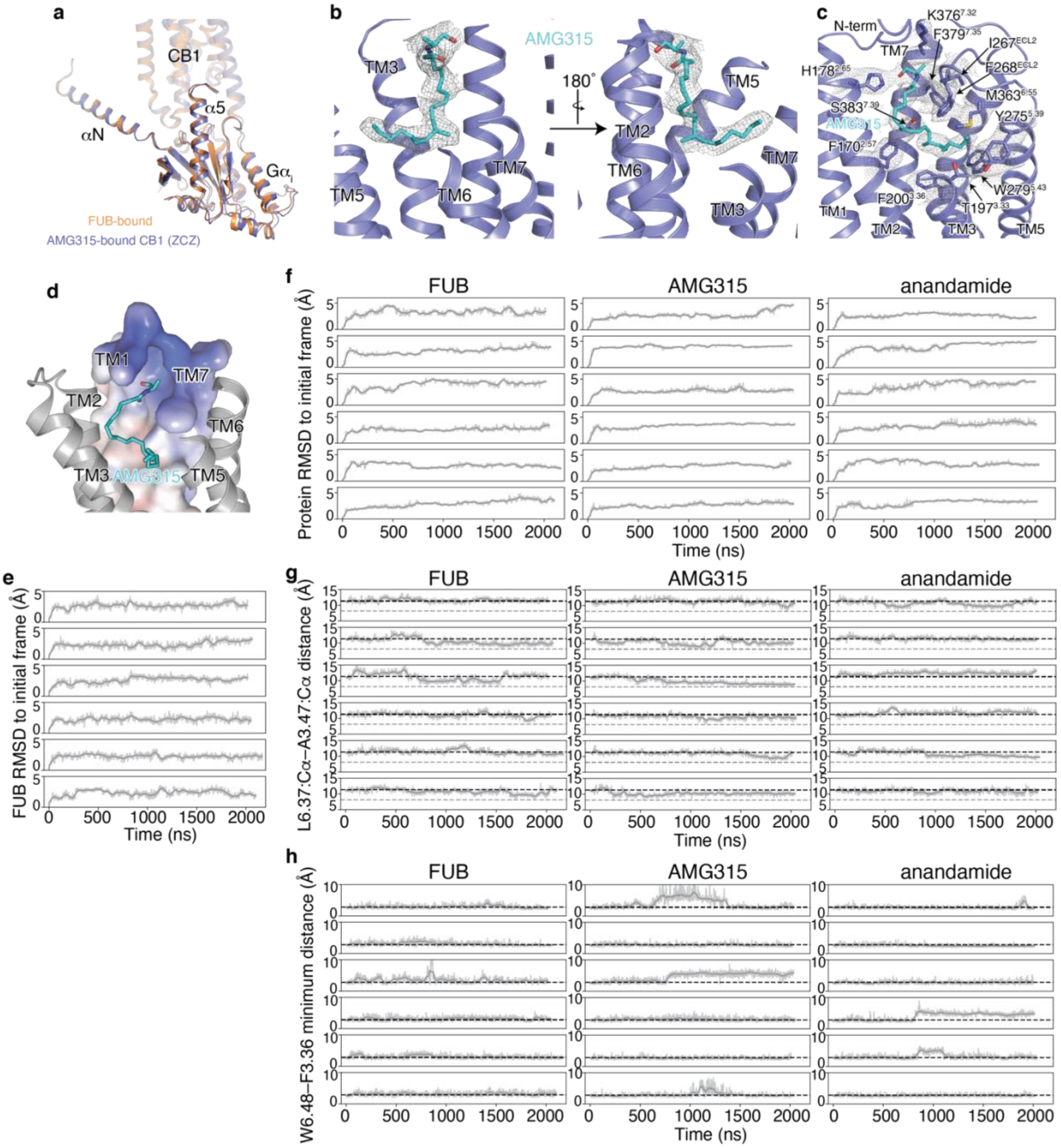

Supplementary Fig.2: AMG315 binding pocket and activation of CB1

a. Overlay of the $G\alpha_i$ subunits from FUB (MDMB-Fubinaca)-bound (orange) and AMG315-bound (blue).
b. Cryo-EM map density of the orthosteric ligand, AMG315 (modeled into the composit map).
c. Surface electrostatics (calculated and analyzed by the APBS Electrostatic *PyMol* Plugin with negative colored blue and positive colored red) of the ligand binding pocket formed by TM1 and TM7.
d. Root-mean-square deviation (RMSD) of FUB (MDMB-Fubinaca) during MD simulations, relative to the initial frame. Thick lines represent moving averages (25-ns averaging window); thin lines represent unsmoothed data. Frames were aligned on non-hydrogen backbone atoms of transmembrane helix (TM) residues (residues 112–144, 150–179, 185–

220, 229–254, 272–312, 332–369, 375–413), and the RMSD calculated on all atoms of the ligand.

e. Protein backbone RMSD (Å) during MD simulations. Frames were aligned on non-hydrogen backbone atoms of TM residues, and the RMSD was calculated on the same atoms.
f. Distance between Cα atoms of residues L6.37 and A3.47 (Å) during MD simulations, used to measure the activation state of the receptor. Black dashed line represents the value of this distance in the experimentally determined active-state structure from which the simulations were initiated. Grey dashed line represents the value in the inactive-state structure (PDB: 5U09 [10.2210/pdb5U09/pdb]).
g. Minimum distance between atoms of residues W6.48 and L3.36 (Å) during MD simulation. Black dashed line represents the value in the active-state structure.

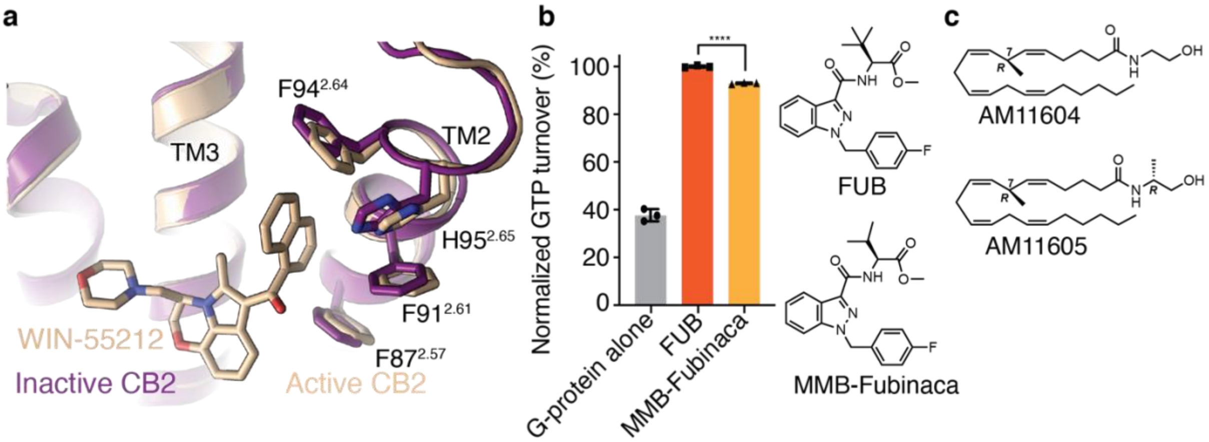

Supplementary Fig.3: Structural changes in CB2 (Cannabinoid receptor 2) on activation and GTP turnover with Fubinaca derivatives

a. Active (PDB: 6PT0 [10.2210/pdb6PT0/pdb]) and inactive (PDB: 6KPC [10.2210/pdb6KPC/pdb]) structures of CB2 (Cannabinoid receptor 2) showing no change in TM2 position upon activation.

b. GTP turnover assay showing more turnover with FUB (MDMB-Fubinaca) compared to MMB-Fubinaca. (Mean ± SD, $p < 0.001$****, unpaired t-test (two-tailed), n=3 independent measurements)

c. Chemical structure of AM11604 and AM11605.

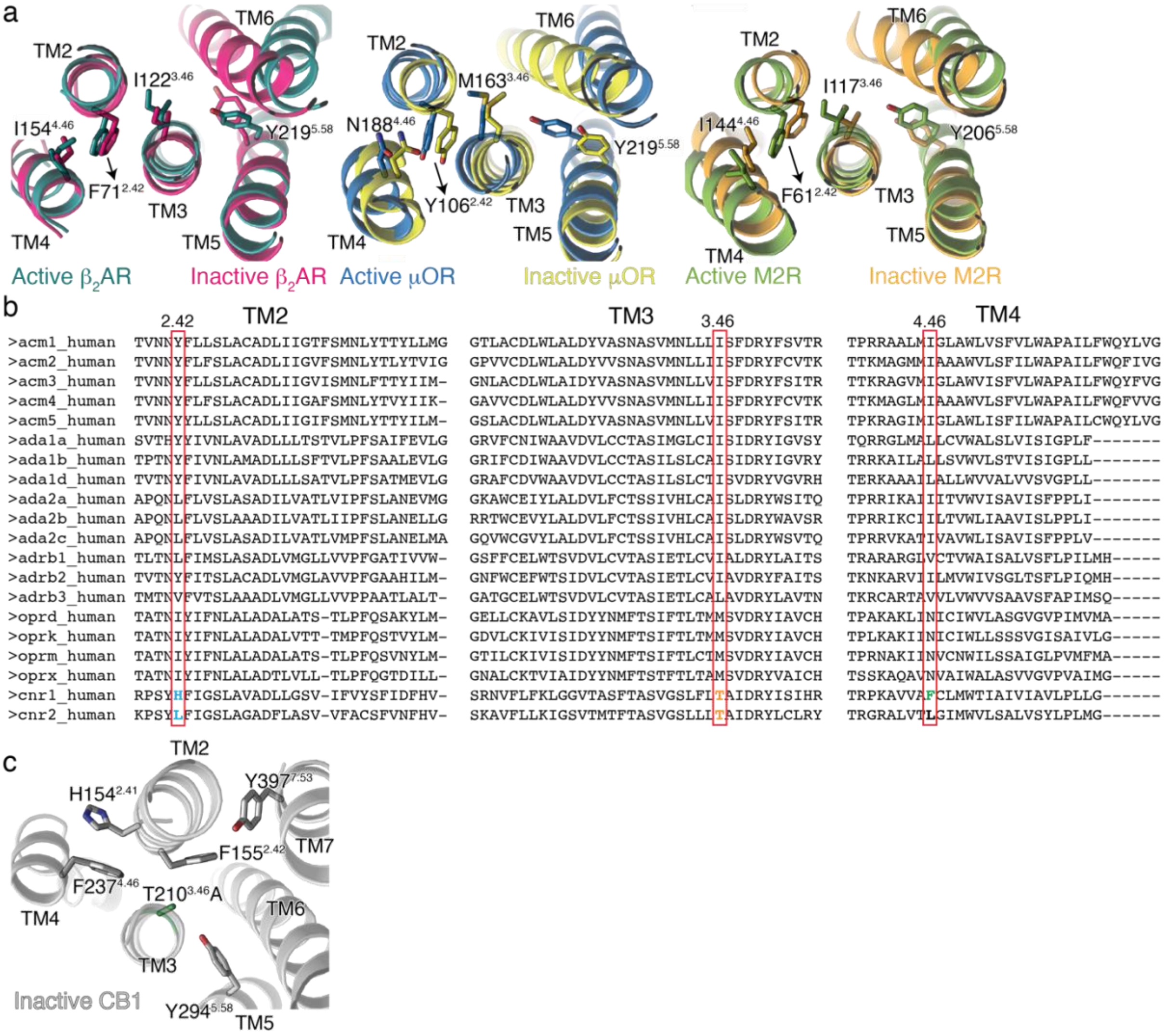

Supplementary Fig.4: Activation of GPCRs

a. Structural changes to TM2-3-4 upon activation in β2AR (β2-adrenergic receptor) (Active, PDB: 3SN6 [10.2210/pdb3SN6/pdb], teal and Inactive PDB: 2RH1 [10.2210/pdb2RH1/pdb], magenta) ,μOR (μ–opioid receptor) (Active, PDB: 6DDE [10.2210/pdb6DDE/pdb], blue and Inactive PDB: 4DKL [10.2210/pdb4DKL/pdb], yellow) and M2R (Muscarinic acetylcholine receptor M2) (Active, PDB: 6OIK [10.2210/pdb6OIK/pdb], green and Inactive PDB: 3UON [10.2210/pdb3UON/pdb], orange).
b. Alignment of GPCRs showing differences in residues at position 2.42, 3.46 and 4.46.
c. In the inactive structure (PDB code 5U09 [10.2210/pdb5U09/pdb], grey), residue 3.46 which is Thr in WT was mutated to Ala (coloured green) to aid in structural determination. This inactivating T210$^{3.46}$A mutation is close to F155$^{2.42}$ and might influence its conformation.

Supplementary Table 1: CryoEM data collection, model refinement and validation

**Data Collection**	**Global Refinement (Local Refinement)**
Voltage (kV)	300
Magnification	x29,000
Total electron dose ($e^-/Å^2$)	80.09
Defocus range (μm)	-1.0 - -2.0
Calibrated pixel size (Å)	0.8521
Micrographs collected	8,332
**Data Processing**	
Extracted particles	2,965,527
Particles used for final reconstruction	530,918
Final map resolution (Å, 0.143 FSC)	2.8 (3.2)
Map resolution range (Å)	2.6-5.0 (3.0-5.0)
Map sharpening B factor ($Å^2$)	116.2 (137.7)
**Model Content**	
Initial models used (PDB code)	6N4B (CB1/G_i/scFV16)
Total number of atoms	8,236
No. of protein residues	1,125
No. of ligands	1
**Model Validation**	
CC map vs. model (%)	81.6
RMSD	
Bond lengths (Å) / Bond angles (°)	0.015 (0.005 / 0.729)
Ramachandran plot statistics	
Favored (%)	89.52 (96.75)
Allowed (%)	10.48 (3.25)
Outliers (%)	0.0 (0.0)
Rotamer outliers (%)	0.0 (0.76)
C-beta deviations	0.0 (0.0)
Clash score	8.76 (3.52)
