# Supplementary material for: Structural basis for activation of CB1 by an endocannabinoid analog
Source: Nat Commun. 2023 May 9;14:2672. doi: 10.1038/s41467-023-37864-4 (PMC10169858; doi:10.1038/s41467-023-37864-4)
Supplement: Supplementary file 2 — Reporting Summary [file 41467_2023_37864_MOESM2_ESM.pdf]

nature portfolio

Corresponding author(s): Alexandros Makriyannis, Georgios Skiniotis, Brian Kobilka

Last updated by author(s): Mar 14, 2023

Reporting Summary

Nature Portfolio wishes to improve the reproducibility of the work that we publish. This form provides structure for consistency and transparency in reporting. For further information on Nature Portfolio policies, see our Editorial Policies and the Editorial Policy Checklist.

Statistics

For all statistical analyses, confirm that the following items are present in the figure legend, table legend, main text, or Methods section.

n/a	Confirmed	
☐	☒	The exact sample size (*n*) for each experimental group/condition, given as a discrete number and unit of measurement
☐	☒	A statement on whether measurements were taken from distinct samples or whether the same sample was measured repeatedly
☐	☒	The statistical test(s) used AND whether they are one- or two-sided<br>*Only common tests should be described solely by name; describe more complex techniques in the Methods section.*
☒	☐	A description of all covariates tested
☒	☐	A description of any assumptions or corrections, such as tests of normality and adjustment for multiple comparisons
☐	☒	A full description of the statistical parameters including central tendency (e.g. means) or other basic estimates (e.g. regression coefficient) AND variation (e.g. standard deviation) or associated estimates of uncertainty (e.g. confidence intervals)
☐	☒	For null hypothesis testing, the test statistic (e.g. *F*, *t*, *r*) with confidence intervals, effect sizes, degrees of freedom and *P* value noted<br>*Give P values as exact values whenever suitable.*
☒	☐	For Bayesian analysis, information on the choice of priors and Markov chain Monte Carlo settings
☒	☐	For hierarchical and complex designs, identification of the appropriate level for tests and full reporting of outcomes
☒	☐	Estimates of effect sizes (e.g. Cohen's *d*, Pearson's *r*), indicating how they were calculated

Our web collection on statistics for biologists contains articles on many of the points above.

Software and code

Policy information about availability of computer code

Data collection	Cryo-EM data was collected automatically on a Titan Krios (FEI) using SerialEM.
Data analysis	FluorEssence v3.8, MotionCor2, CTFFIND4, RELION 3.1, cryosparc 3.3.1, phenix 1.19.2, UCSF Chimera 1.15, Coot 0.8.9, Maestro 5.0, AMBER18, GraphPad Prism 9, PyMOL 2.5.4

For manuscripts utilizing custom algorithms or software that are central to the research but not yet described in published literature, software must be made available to editors and reviewers. We strongly encourage code deposition in a community repository (e.g. GitHub). See the Nature Portfolio guidelines for submitting code & software for further information.

Data

Policy information about availability of data

All manuscripts must include a data availability statement. This statement should provide the following information, where applicable:

- Accession codes, unique identifiers, or web links for publicly available datasets
- A description of any restrictions on data availability
- For clinical datasets or third party data, please ensure that the statement adheres to our policy

Electron Microscopy Data Bank (EMDB) under accession codes EMD-40052 [https://www.ebi.ac.uk/emdb/search/EMD-40052], EMD-40057 [https://www.ebi.ac.uk/emdb/EMD-40057] and EMD-40058 [https://www.ebi.ac.uk/emdb/EMD-40058]. Model coordinates from this manuscript have been deposited in the Protein Data Bank (PDB) under accession number 8GHV [10.2210/pdb8ghv/pdb].

Previously published structures mentioned in this paper can be accessed via the following PDB codes:
PDB: 6KQI [10.2210/pdb6KQI/pdb]
PDB: 5U09 [10.2210/pdb5U09/pdb]
PDB: 6PT0 [10.2210/pdb6PT0/pdb]
PDB: 6KPC [10.2210/pdb6KPC/pdb]
PDB: 3SN6 [10.2210/pdb3SN6/pdb]
PDB: 2RH1 [10.2210/pdb2RH1/pdb]
PDB: 6DDE [10.2210/pdb6DDE/pdb]
PDB: 4DKL [10.2210/pdb4DKL/pdb]
PDB: 6OIK [10.2210/pdb6OIK/pdb]
PDB: 3UON [10.2210/pdb3UON/pdb]
PDB: 5U09 [10.2210/pdb5U09/pdb]
PDB: 6N4B [10.2210/pdb6N4B/pdb]
PDB: 5XRA [0.2210/pdb5XRA/pdb]

Human research participants

Policy information about studies involving human research participants and Sex and Gender in Research.

Reporting on sex and gender	Not Applicable
Population characteristics	Not Applicable
Recruitment	Not Applicable
Ethics oversight	Not Applicable

Note that full information on the approval of the study protocol must also be provided in the manuscript.

Field-specific reporting

Please select the one below that is the best fit for your research. If you are not sure, read the appropriate sections before making your selection.

☒ Life sciences ☐ Behavioural & social sciences ☐ Ecological, evolutionary & environmental sciences

For a reference copy of the document with all sections, see nature.com/documents/nr-reporting-summary-flat.pdf

Life sciences study design

All studies must disclose on these points even when the disclosure is negative.

Sample size	Sample size were not predetermined. For Cryo-EM, 8,332 micrographs were collected, which was enough to obtain a 2.8 Å map. For the functional data atleast three independent replicates were carried out, which was enough to perform statistical analysis.
Data exclusions	No data was excluded from analysis.
Replication	Atleast three independent experiments were conducted for the functional assays. All attempts at replication were successful.
Randomization	No randomization was performed as data was collected without the necessity for choosing.
Blinding	Since no subjective assessment was required, blinding was not required.

Reporting for specific materials, systems and methods

We require information from authors about some types of materials, experimental systems and methods used in many studies. Here, indicate whether each material, system or method listed is relevant to your study. If you are not sure if a list item applies to your research, read the appropriate section before selecting a response.

Materials & experimental systems

n/a	Involved in the study
☐	☒ Antibodies
☐	☒ Eukaryotic cell lines
☒	☐ Palaeontology and archaeology
☒	☐ Animals and other organisms
☒	☐ Clinical data
☒	☐ Dual use research of concern

Methods

n/a	Involved in the study
☒	☐ ChIP-seq
☒	☐ Flow cytometry
☒	☐ MRI-based neuroimaging

Antibodies

Antibodies used	Single chain Fv16 (scFv16) was used to stabilise the CB1-Gi complex. It is described in Maeda et al., Nature Comm, 2018. Initially the antibody version of scFv16 was obtained from mouse, as described in the paper mentioned above. Since the sequence is known, the scFv16 was produced in Trichuplusia ni (Hi-5) cells. scFv16 is used to stabilise the CB1-Gi complex and is not used for detection. It is used in the molar ratio of 1:2 (Gi1:scFv16) during complex formation.
Validation	Since scFv16 is not produced in the Kobilka Lab and is used for probing or detection, no validation is needed.

Eukaryotic cell lines

Policy information about cell lines and Sex and Gender in Research

Cell line source(s)	Spodoptera frugiperda (Sf9), Expression Systems, Cat no. 94-001S.<br>Trichuplusia ni (Hi5), Expression Systems, Cat no. 94011S<br>HEK293A, Thermo Fisher Scientific, Cat no. R70507
Authentication	Cell lines are maintained by the supplier. No additional authentication was performed by the authors of this study
Mycoplasma contamination	Cell lines are tested by manufacturer for contamination, but not were not further tested by the authors of this study.
Commonly misidentified lines (See ICLAC register)	No commonly misidentified lines were used in this study.
